# Mandibular distraction osteogenesis in patients with unilateral TMJ involvement in juvenile idiopathic arthritis

**DOI:** 10.1186/1546-0096-9-S1-P177

**Published:** 2011-09-14

**Authors:** SE Nørholt, TK Pedersen, T Herlin

**Affiliations:** 1 Department of Orthognathic Surgery, Aarhus University Hospital, Denmark; 2 Department of Orthodontics, Faculty of Health Science, University of Aarhus, Denmark; 3 Department of Pediatrics, Aarhus University Hospital, Denmark

## Background

In juvenile idiopathic arthritis (JIA) the temporomandibular joint is frequently involved which may result in severe growth impairment of the mandible. In cases of unilateral involvement growth impairment will result in an asymmetric lower face.

## Aim

To evaluate long-term effect of distraction osteogenesis (DO) in JIA patients with unilateral TMJ in a prospective study.

## Methods

Twenty-three patients with JIA (mean age 16 y (11-34) underwent unilateral DO. Patients were treated according to standardized protocol: preoperative orthodontic planning, cephalometric analysis calculating distraction vector, and transfer of vector to the surgical procedure. An occlusal splint was used full-time. Data were ascertained before DO, after removal of distraction device, 6 and 12 months after DO, and at latest follow-up visit. Frontal cephalograms and orthopantomograms were performed comparing normal and affected side.

## Results

TMJ function was within normal ranges before DO and with no significant changes after in all except two patients. Pain related to TMJ loading as a result of arthritis was reduced after treatment (5 before, none after). Mandibular asymmetry was corrected, relaxed lip closure was improved and respiratory pattern normalized. Tongue dysfunction was present in eight patients before treatment and in two patients at follow-up. Dental occlusion was normalized for all but two patients. Before treatment the maximum jaw opening was 47.6 mm with normal laterotrusion and protrusion. Shortly after DO mandibular mobility was significantly reduced but during follow-up it normalized.

## Conclusions

In JIA patients with unilateral TMJ arthritis DO resulted in good skeletal and occlusal stability. Good function of the joints was preserved at long-term follow-up. The affected joint should be guarded by use of an occlusal splint during and after the distraction process until occlusion has been established.

